# Family history assessment for colorectal cancer (CRC) risk analysis - comparison of diagram- and questionnaire-based web interfaces

**DOI:** 10.1186/s12911-015-0211-1

**Published:** 2015-11-18

**Authors:** Michael Schultz, Steven Bohwan Seo, Alec Holt, Holger Regenbrecht

**Affiliations:** Department of Medicine, Dunedin School of Medicine, University of Otago, PO Box 56, Dunedin, 9054 New Zealand; Gastroenterology Unit, Southern District Health Board, Dunedin, New Zealand; Department of Information Science, University of Otago, Dunedin, New Zealand

**Keywords:** Colorectal cancer, Family history, Surveillance, Health systems, Health informatics, Human-computer interaction, Gastroenterology

## Abstract

**Background:**

Colorectal cancer (CRC) has a high incidence, especially in New Zealand. The reasons for this are unknown. While most cancers develop sporadically, a positive family history, determined by the number and age at diagnosis of affected first and second degree relatives with CRC is one of the major factors, which may increase an individual’s lifetime risk. Before a patient can be enrolled in a surveillance program a detailed assessment and documentation of the family history is important but time consuming and often inaccurate. The documentation is usually paper-based. Our aim was therefore to develop and validate the usability and efficacy of a web-based family history assessment tool for CRC suitable for the general population. The tool was also to calculate the risk and make a recommendation for surveillance.

**Methods:**

Two versions of an electronic assessment tool, diagram-based and questionnaire-based, were developed with the risk analysis and recommendations for surveillance based on the New Zealand Guidelines Group recommendations. Accuracy of our tool was tested prior to the study by comparing risk calculations based on family history by experienced gastroenterologists with the electronic assessment. The general public, visiting a local science fair were asked to use and comment on the usability of the two interfaces.

**Results:**

Ninety people assessed and commented on the two interfaces. Both interfaces were effective in assessing the risk to develop CRC through their familial history for CRC. However, the questionnaire-based interface performed with significantly better satisfaction (*p* = 0.001) than the diagram-based interface. There was no difference in efficacy though.

**Conclusion:**

We conclude that a web-based questionnaire tool can assist in the accurate documentation and analysis of the family history relevant to determine the individual risk of CRC based on local guidelines. The calculator is now implemented and assessable through the web-page of a local charity for colorectal cancer awareness and integral part of the local general practitioners’ e-referral system for colonic imaging.

## Background

Colorectal cancer (CRC) is one of the most incident malignant tumours in the developed world and also in New Zealand (NZ) where it ranks second for incidence and mortality [1]. Several campaigns have been developed to increase public awareness but one has to differentiate between symptomatic patients, those with so-called red flags [2] and asymptomatic patients. These latter patients constitute the general population as well as a particular group, based on their family history with an increased risk of developing CRC. These patients are most often not aware of their individual risk to develop CRC due to their family history.

New Zealand does not yet have a population-wide screening program but surveillance of patients with a high risk of developing CRC is offered within the public health system. Most colorectal cancers develop spontaneously but a positive family history is one of the major factors, which may increase an individual’s lifetime risk [3]. The New Zealand Guidelines Group (NZGG), later replaced by the Colorectal Cancer Working Group instituted by the New Zealand Ministry of Health published an analysis of the individual risk for the development of CRC in the asymptomatic NZ population. This is primarily based on the number and age at diagnosis of affected first and second degree relatives with CRC. It also takes into account a personal history such as Inflammatory Bowel Disease, previous polyps, personal history of cancers, the possibility of the presence of Lynch syndrome or the belonging to a family fulfilling the Amsterdam criteria, etc. Based on these criteria a patient is entered into an endoscopic surveillance program [4]. To identify these patients, traditionally we used a paper-based questionnaire to collect and analyse a patient’s risk of developing CRC based on the individual’s family history. However, completion of the family history questionnaire and analysis is time consuming and the risk prediction depends on the accuracy of the entered data as well as on the physician/specialist nurse to analyse. As recommendations for screening and surveillance of patients with an increased risk of CRC are solely based on the family history in asymptomatic patients this is a crucial first step to enter a screening program if indicated. Previous research, for instance in the estimation of the risk to develop coronary heart disease (CHD) has shown that providers do not accurately estimate the risk on their own [5].

Web-based calculators to estimate the risk for different diseases are common and mostly used for cardiovascular disease [6], skin cancer, breast cancer and others (e.g. [7]). These calculators are usually designed for the general public and combine protective and harmful effects of different factors (diet, physical activity, weight, ethnicity, etc.) to estimate the risk [8]. However, often (like in New Zealand) access to screening and surveillance for CRC is only funded for symptomatic patients or those with an increased risk based on very specific and well established criteria such as the family history. Regarding CRC, the contribution of the family history amongst all different risk modulating factors is best researched and well accepted [2]. We therefore aimed our focus in the first instance to develop a web-based tool to identify patients at risk to develop colorectal cancer based on their family history.

**Fig. 1:**
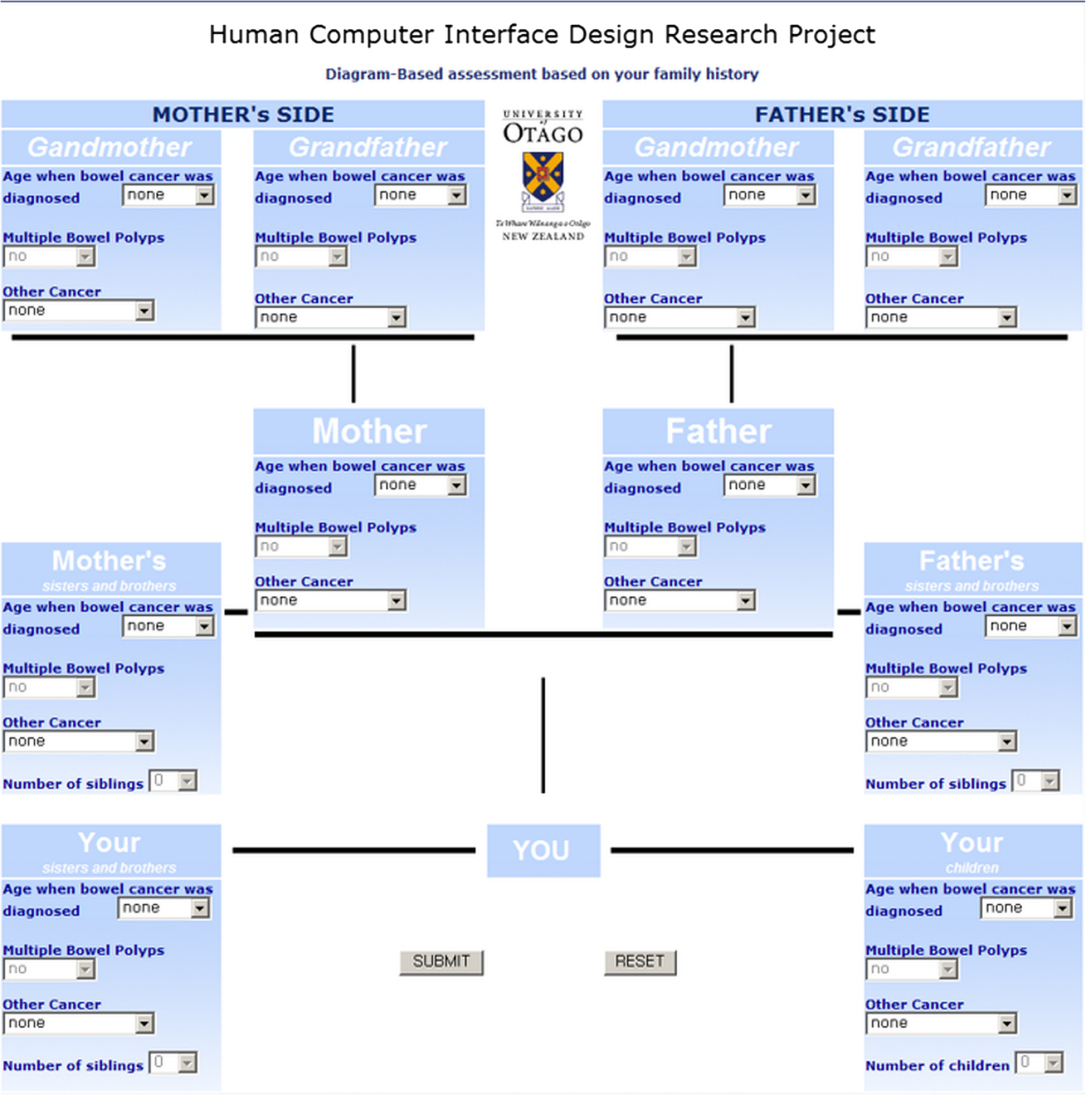
Screenshot of the diagram-based interface of the CRC risk assessment tool utilizing the opportunities of graphical user interfaces (GUI) elements

**Fig. 2:**
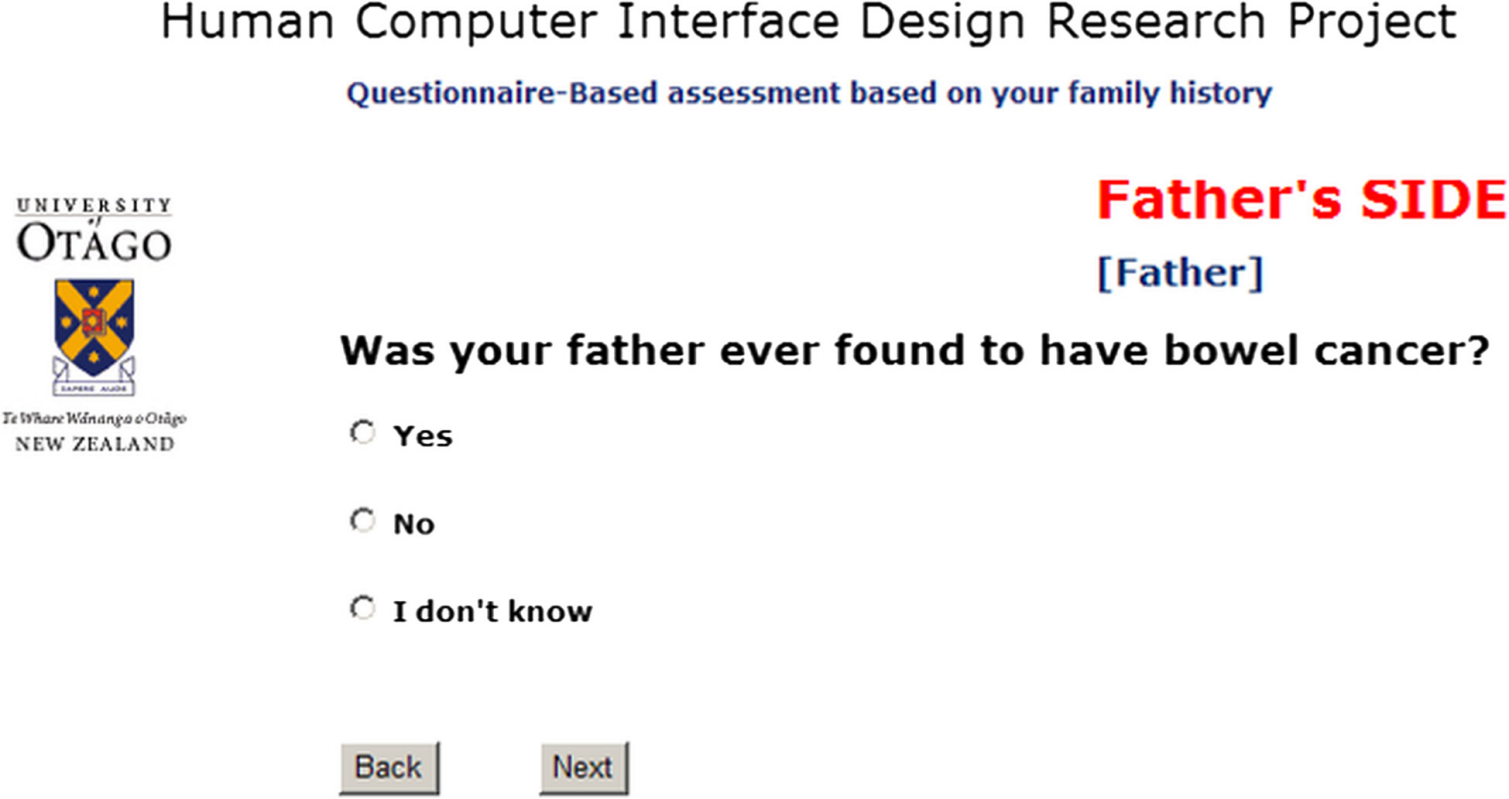
Screenshot of the questionnaire-based interface of the CRC risk assessment tool mimicking the standard paper-based questionnaire as close as possible

Beuscart-Zéphir et al. [9] stated that a poorly designed clinical system can lead to usability problems and may disrupt the normal flow of activities. Following development of an algorithm, we aimed to study usability of two interfaces (questionnaire-based v diagram-based, see Figs. 1 and 2) to decide which version of the interface should be used - as usability is the key attribute for introducing information systems into medical fields [10]. The diagram-based version utilizes the opportunities of graphical user interfaces (GUI) elements, while the questionnaire-based interface mimics the standard questionnaire as close as possible. To date it remains unclear which of the two approaches is more beneficial for end users, however, uptake of this tool and with it improvement of the process of documentation and analysis of the family history depends crucially on the usability being evaluated with the general public in mind.

House et al. [11] mailed printed hard copies of CRC family history questionnaires with great success in 1999. While Emery at al. [12] developed and used a web-based system for health professionals (GRAIDS pedigree) and Acheson et al. [13] developed a phone-based system (GREAT) where the users operate a telephone keypad system and administrators enter the data into the system. Both approaches have been reported as acceptable forms of gathering information but analysis was left to the health providers.

Westman et al. [14] developed and tested a touchscreen-based system in public space (hospital) which used a decision-point-based GUI structure, but unfortunately, 95 % of the users felt uncomfortable using the system. Yoon et al. [15] developed an effective self-administered, web-based, family history tool for 6 diseases, including colorectal cancer, following a questionnaire approach. We too used a questionnaire approach for a mobile Inflammatory Bowel Disease monitoring program with success [16].

In a more recent study Vogel et al. [17] found that the application of a self-administered family history questionnaire to identify women for referral increased recognition of patients appropriate for genetic counselling. A web-based family history assessment questionnaire was used in Baer et al.’s study [18] and the authors demonstrated good general feasibility as well as the successful link into electronic health records.

Murray et al. [19] compared an interactive voice response system (IVR), an internet portal, and a waiting room portal access computer for patient-entered family health history. They found that electronic, patient-entered data can be obtained at higher rates than standard-care provider-entered data. However, they state that further research is needed on matching different portals to patient preference.

Recent research by Dekker et al. shows [20] that easy-to-use, online family risk assessment tools can detect patients effectively and efficiently. However, it remains unclear what the right form of interface for such a tool should be.

Given the ambivalent outcomes of the different approaches (questionnaires versus other forms) we decided to develop both versions and to compare them: questionnaire-based v diagram-based.

## Methods

The overall aim of this study was to improve information gathering on the family risk contributing to the development of colorectal cancer and to aid with the analysis of this information.

The first aim was to develop a web-based tool (incl. assessment algorithm) to gather family history information and to analyse this to make a recommendation regarding necessary surveillance.

The second aim was to evaluate different interfaces according to their effectiveness and suitability to gather the necessary information.

### Construction of the assessment algorithm

**Fig. 3:**
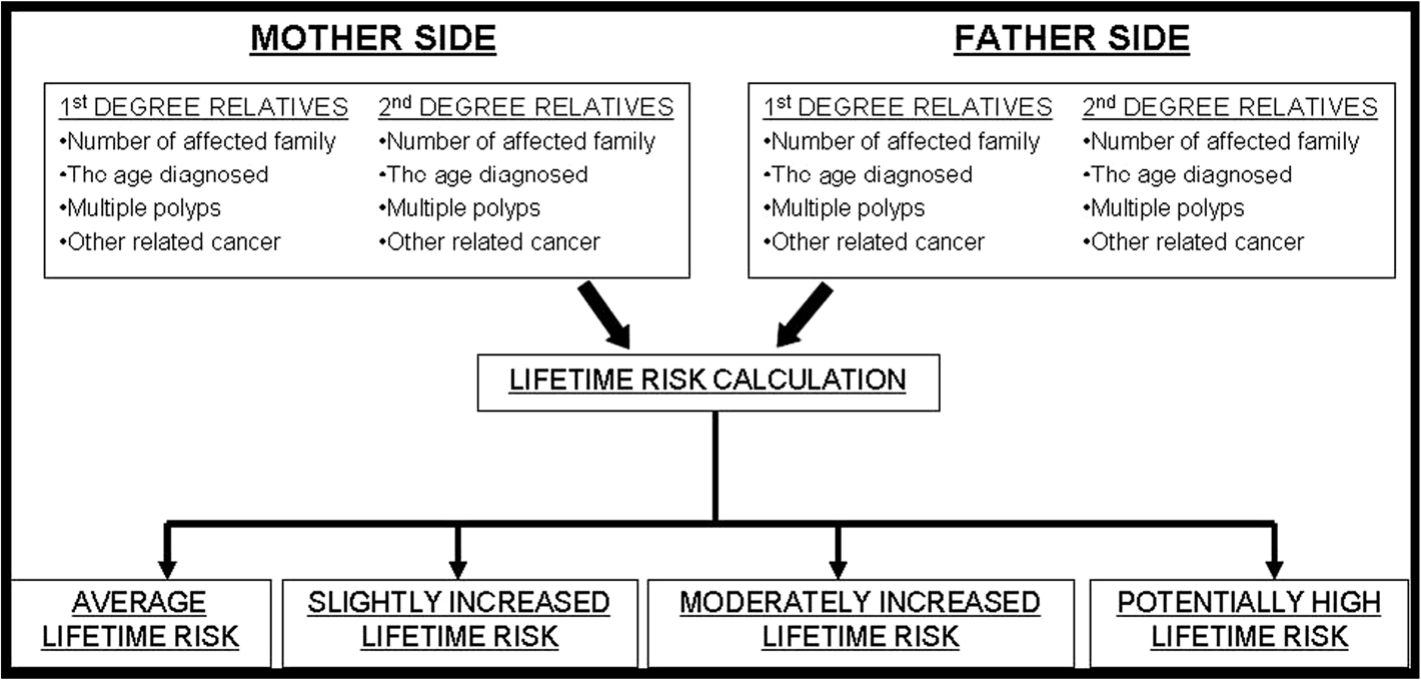
Conceptual algorithm for the family history assessment. The information entered by the user was calculated by the “Lifetime Risk Calculation” and one of four categories defined by the guideline was given to the user with corresponding recommendations

In this study an assessment algorithm was constructed in the cooperation specialist physicians (gastroenterologists) and informatics experts. Initially, as shown in Fig. 3, the algorithm grouped the first and second degree relatives into two sides, mother and father. The information entered by the user was calculated by the “lifetime risk calculation” and one of four categories defined by the NZGG guideline was given to the user with corresponding recommendations. After the “lifetime risk calculation” analysed the number of affected family members, the age diagnosed, multiple polyps, and other related cancers, it determined which category the user was included in. The NZGG guideline defines several rules to determine risk categories (average lifetime risk, slightly increased risk, moderately increased risk and potentially high risk) and the gastroenterologists translated the risk levels and recommendations to help the general population conveniently understand these.

The algorithms basically consists of:

1. family history information collection,
2. grouping of family history into three family sides (mother, father, and you),
3. classification of the information into two degrees of relatives, First Degree Relatives (FDR) and Second Degree Relatives (SDR), and the
4. lifetime risk calculation.

The algorithm groups the information into three sides of the family, and each side of the family history information is then classified into FDR and SDR:

- The “mother’s side” is composed of the mother, maternal grandmother and grandfather, mother’s siblings and their children (nieces and nephews). The mother is the FDR and the others are considered SDR.
- The “father’s side” is composed of the father, paternal grandmother and grandfather, father’s siblings and their children (nieces and nephews). The father is the FDR and the others are considered SDR.
- “Your side” is composed of the user’s siblings and children, both of them as FDR.

Finally, the “lifetime risk calculation” algorithm interprets and counts the classified information to determine which category the user should be included in.

The actual web-based tool was implemented using Active Server Pages - ASP, extended Hypertext Markup Language - XHTML, and JavaScript (with Cookies).

We developed two interfaces (diagram-based and questionnaire-based) for comparison of satisfaction and efficiency.

### Diagram based interface development

The diagram-based interface (Fig. 1) was inspired by a revised paper-based family history tree diagram, which was recommended by the New Zealand Guidelines Group, and it is still being used to examine affected first and second degree relatives.

With a paper-based family tree, gastroenterologists can easily see the whole familial history of an individual at a glance and perform the assessment easily. The diagram-based interface displayed the family history tree on only one page of the web browser and first and second degree relatives were presented as small blocks on the top, such as mother, grandfather, your children and so on. It is relatively easy to recognize where the relevant information should be entered within the blocks for each respective relative.

### Questionnaire based interface development

The questionnaire-based interface (Fig. 2) follows a sequential approach presenting one question at a time. The questions comprised open field questions and radio buttons relating to when the cancer was diagnosed, whether the relative had multiple polyps and had other cancers before. Large font size headings have been added above each question on the page for better navigation (side of family tree etc.).

### Usability evaluation

A usability study was conducted to evaluate the efficiency and user satisfaction of these two interface modes, diagram-based and questionnaire-based interfaces.

#### Study variables

The independent variable in the study was the two modes of interface; diagram-based and questionnaire-based. These variables are defined as follows:

- Diagram-based interface: The interface which was developed from a family history tree in evidence-based best practice guidelines.
- Questionnaire-based interface: Every single question was presented on a single page.

The dependent variables in the study were efficiency and satisfaction. These variables are defined as follows:

- Efficiency: the time it takes to complete the assessment task used in this study with the respective interface. The most efficient interface was the one with the least time spent to complete the task.
- Satisfaction: the user’s subjective rating of freedom from discomfort and their positive attitudes towards the use of each interface in the experiment. User satisfaction was measured with a questionnaire in this study.

Potential confounding variables have been identified in this study and steps have been taken to minimize their effects. All subjects were randomly but evenly divided into two groups. Subjects who had experience of the diagram or similar assessment were monitored in a demographic survey and were excluded from this study.

Other potentially confounding variables which might have affected this study were gender, age and physical disabilities. Simon determined that there were differences between gender-based perception and satisfaction with the use of a web browser [21] and there is evidence to suggest that older adult users may encounter web barrier associated with normal aging [22]. Therefore, gender and age were screened in the demographic survey and analysed for any potential effects on the results in this study (none found). Physical disabilities may also affect a subject’s performance, such as uncorrected vision impairment and physical disabilities of fingers, wrist, arm, shoulders and/or neck, however no participants with these disabilities were identified with the demographic survey questionnaire.

#### Subjects

Participants voluntarily came to the experiment booth or were invited by the gastroenterology department staff after a casual consultation at the festival event.

Participants were given a participant information sheet explaining the purpose of the study (Usability analysis of Human-Computer Interface for colorectal cancer risk assessment based on family history) and the experimental procedure. An attached consent form was also provided to explain participant rights and anonymity and each participant was asked to read and sign before proceeding with the experiment. After that, the participant demographic questionnaire was completed by the participant. Whenever the participant asked a question during the experiment, the experimenter answered and made notes on any details of the situation.

After the participant finished the assessment and clicked the “submit” button the experimenter recorded the time displayed on the summary page. While the participant was asked to fill in the after scenario questionnaire a result and summary page was printed and given to the participant as a token of appreciation. This summary page consisted of a summary of the entries, the calculated risk and a recommendation to see their general practitioner to discuss surveillance.

Ninety subjects (41 male) from a Science Festival event in Dunedin, New Zealand volunteered to participate in this study. The subjects were in various ranges of age groups, under 18 years (8), 18–24 years (9), 25–34 years (9), 35–44 years (29), 45–54 years (21), 55¬64 years (9) and over 64 years (5). 88 % of the subjects were experienced with web browsers, with at least once a week to use the Internet and the rest of them used the Internet less than once a month (11), once a month (2) and several times a month (7).

#### Study design

##### Task

The task scenario used in this study was an actual family history assessment for CRC that an individual might make use in a real world situation.

While beginning the task was from identical introduction and instruction pages the two interfaces where quite different from thereon:

- Diagram-based interface: A subject could select one of several options from the drop-down boxes and each first and second degree relative information was provided on a single web page. A “Reset” button could be pressed to reset all information entered with a confirmation message. After entering all information on the single page, the “Submit” button had to be pressed to finish the risk assessment and to obtain a printed risk level and recommendation sheet.
- Questionnaire-based interface: Only one question regarding first or second degree relatives was presented on each web page. A “next” button had to be pressed to go to the next question. When the final question was answered, a “submit” button appeared to complete the task and to obtain the risk level and recommendation sheet.

While each interface of the assessment had different steps and procedures to obtain family history they resulted in identical summary pages.

##### Questionnaires

User satisfaction is a user’s subjective response when using a specific product and it is an important correlate of motivation to use a product. It was measured with an after scenario satisfaction questionnaire.

The seventeen questions were introduced from the IBM Computer System Usability Questionnaire (CSUQ) [23] measured on 7-point Likert scales, with anchors of 1 for strongly agree and 7 for strongly disagree for each question. Subjects could also use free text fields to comment. An after scenario questionnaire “assessment of attitude” was used to measure the user satisfaction for the two different interfaces of the assessment tool. This questionnaire comprises questions on the ease of use, ease of task completion, learnability, and interface comprehension amongst others.

In addition to an after scenario questionnaire a participant demographic questionnaire collected subjects’ general information such as age group, gender, any disability, level of familiarity with a family history diagram, and experience with web browsers and online medical assessments.

##### Experimental design

This study used 90 participants (sample estimate of 2x40 [24] plus allowance for missing data) in a between-subjects experimental design. With this design any differences may be overshadowed by differences in the makeup of the test groups so random assignment was conducted to mitigate this effect. All of the participants were pseudo-randomly assigned (2x45 prepared labels were randomly picked from by the experimenter for each participant arriving) to one of the two groups of 45 subjects per group (diagram-based vs questionnaire-based interface) and the subjects of each group performed the family history assessment task with the interface allocated.

##### Assumptions

There are several assumptions supporting this study:

- The family history assessment tool entirely follows evidence-based best practice guidelines and the feasibility testing of the algorithm is successfully done by the gastroenterologists so that a use of the assessment tool can be practically applied to real world situations.
- The assessment is performed anonymously and the tool does store any information entered or derived only for the purpose of experimental data analysis.
- The questions used in this study are suitable to measure participants’ subjective satisfaction with each interface.
- Subjects participated in this study are representative of the general population.

##### Statistical analysis

Data analysis was performed with SPSS version 15.0 and all significance testing was performed at the 95 % confidence interval. An independent groups t-test was performed and the data collected from all 90 participants was used in this analysis. A Mann–Whitney Test was performed to detect differences between the two interfaces while the Little’s MCAR test was used to test for randomness of missing data.

An alpha level of 0.05 was used for statistical testing.

Negatively worded scale items in the after-scenario questionnaire were recoded before the data were analysed.

### Ethical consideration

This project was considered and approved by the University of Otago human Ethics Committee.

## Results and discussion

We developed a web-based tool for identifying an individual’s risk of developing colorectal cancer based on the person’s family history. We compared a diagram-based interface and a questionnaire-based interface of this tool. The analysis of the risk and the subsequent recommendation is based on surveillance recommendations published by the New Zealand Guidelines Group and are therefore sanctioned by the Ministry of Health. In contrast to other available colorectal cancer risk calculators, our calculator uses well defined and validated risk factors for the calculation of the risk. The usability of such a new technology is crucial and we therefore undertook a comparison between two interface modes.

None of the subjects had experience with a family history diagram or anything similar for CRC assessment purposes prior to this experiment while one subject had used a medical assessment tool via the Internet. No participant asked to make corrections of their entries following the review of the summary page.

### Efficiency evaluation

The participants who performed the assessment with the diagram-based interface took longer time (M = 89.2, SD = 7.05) to complete the assessment than did participants with the questionnaire-based interface (M = 80.89, SD = 5.19). An independent groups t-test found a non-significant difference between the mean time taken by each group of participants (*p* = 0.526).

**Fig. 4:**
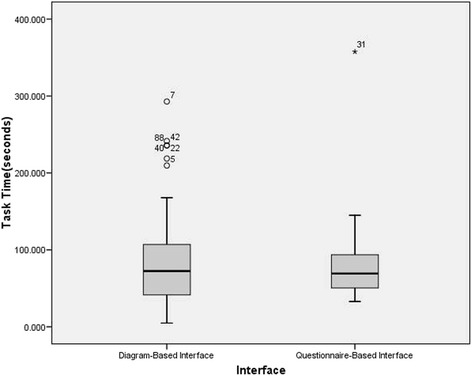
Box plot of task time results for the two interface modes. The time difference was not significant (*p* = 0.771)

Figure 4 shows that the distribution of the task time results for each interface. The results for the diagram-based interface are approximately normal in distribution while the results for the questionnaire-based interface are slightly skewed.

According to normality test for the task time of the two interface mode, the assumption of normality was violated (SPSS advices to use the Shapiro-Wilk test when sample sizes are below 50, *p* < .05), so a Mann–Whitney Test was performed to confirm that there was no significant difference between the two interfaces (*p* = 0.771). No significant difference on the task time was found in the efficiency of the diagram-based and questionnaire-based interface.

Therefore, the questionnaire-based interface did not have better efficiency than the diagram-based interface. A reason for this finding might be found in the information presentation structure of the questionnaire-based interface. While the diagram-based interface had only one web page to enter related information, with the questionnaire-based interface there were at least eleven pages to fill in and even forty five at most depending on how extensive the family history was. The structure required the subjects to put more effort (i.e. more mouse clicking and reading the pages) on the assessment so that the task time could not be efficient in the end.

### Satisfaction evaluation

Twenty-one study participants did not answer a few questions, so missing value analysis was required to deal with the missing data. In 1988 Little’s chi-square statistic was introduced to test whether missing values were completely random [25]. The Little’s MCAR test indicated that the data missing was completely at random (*p* = 0.837). This confirms that the probability of missing values was unrelated to the value of any other variables in this study so that a single new data set that has no missing values can be imputed.

The after scenario questionnaire consists of seventeen items to assess users’ satisfaction and these items were summed to create a total score from each study participant with a Cronbach’s Alpha of 0.901.

The average satisfaction score of the questionnaire-based interface was significantly higher than the score for the diagram-based interface (*p* = 0.001).

The cumulative score of all satisfaction items (maximum of 8x17 = 136) for the diagram-based interface (M = 101.12, SD = 11.9) was lower than the one for the questionnaire-based interface (M = 108.79, SD = 9.08).

**Fig. 5:**
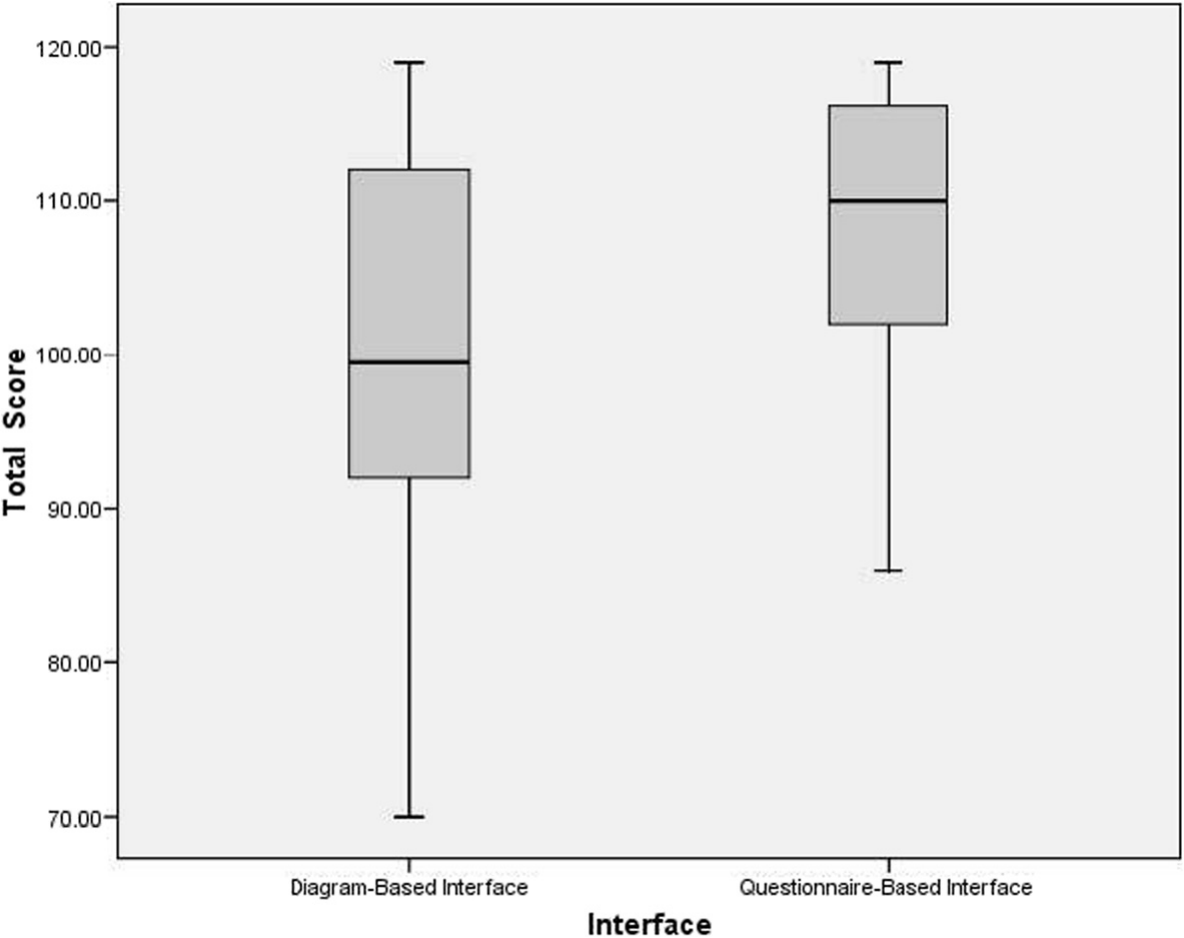
Box plot of the average satisfaction score of both interface modes. The satisfaction score for the questionnaire-based interface was significantly higher than the score for the diagram-based interface (*p* = 0.001)

Figure 5 shows the distribution of the results for satisfaction with each interface. The results for the two interfaces are considerably skewed.

Because the assumption of normality for the data for the diagram-based interface was violated, a Mann–Whitney Test was performed to confirm that there was a significant difference of the results for satisfaction between the two interfaces (*p* = 0.002).

The total score of user satisfaction shows that the questionnaire-based interface was found to be more satisfying to the user than the diagram-based interface; there was a significant difference between the total scores of the two interface modes.

We found that the satisfaction level was significantly higher on the questionnaire-based interface and this can be supported by one of the further findings; the questionnaire-based interface could provide more intuitive and comprehensible ideas with the subjects using written questionnaires while the diagram-based interface required them to interpret and understand the implicit diagram before entering related information.

Several studies were conducted in the past exploring novel methods of gathering health information [11–13]. However, our device is novel in that is analyses data and not only gathers data [11–13]. It also seems important how and where the data gathering tool is and can be accessed as potentially sensitive private clinical data is entered [14]. A web-based tool, even if the access cannot be fully controlled, provides ease of use and access as the findings by Baer, Dekker and Vogel [17, 18, 20] suggest. In contrast to findings by Simon et al. [20] and Becker et al. [21] we did not find differences in gender or sex concerning efficacy or satisfaction.

### Further findings

In the after scenario questionnaire, the question asking about error messages popping up during the completion of the task was not answered by 12 subjects carrying out the questionnaire-based interface and 8 subjects with the diagram-based interface. However, comments made by 19 subjects out of 20 subjects state that they did not see any error messages during the family history assessment and the assessment tool clearly responded to the users. Only one subject did not provide any comment. This finding implies the effectiveness of the family history assessment which was not explicitly measured in the study. Observations showed no obvious errors were made and that every participant successfully completed the assessment.

With the questionnaire-based interface one subject out of forty-five reported that the interface repeated similar styles of question for different first and second degree relatives and the subject did not realize which questions were for whom in the middle of the experiment because he did not see the headings. He had to revert back to the first question and start over answering the questions to get on the right track of the assessment so the total task time became exceptionally longer than for other subjects.

During the observation of the experiment seven subjects out of forty-five who performed the assessment with diagram-based interface had difficulties understanding the diagram during the experiment. They asked several questions in regards to how to read and interpret the diagram so the task time became longer than for other subjects.

### Limitations

There are several limitations to this study. To calculate the level of risk of developing colorectal cancer, we used the recommendations as published by the New Zealand Guidelines Group. These recommendations provided clear scenarios and grouped them into three distinct risk categories. As such, this particular calculator cannot be used for a similar purpose outside New Zealand. However, the main aim for this study was to examine the satisfaction and efficiency of two interfaces and as such our results are generalizable. A further limitation is the setting in which the testing took place. This was artificial and allowed participants to ask questions during the data entry. It remains unclear how many participants would have not continued with the task if on their own. Lastly, more detailed information on usability could have been gathered on the ease of learning and by recording quotes.

## Conclusion

Our findings suggest that users should be presented with interfaces which match their prior experience and that new interfaces should be introduced in an iterative and incremental way building on the known experiences of the users.

One of the major advantages of this web-based tool is that recommendation are given in a timely manner and reflect the funding situation in New Zealand in that a recommendation for surveillance will be acted upon. This is the main difference to other calculators using less robust risk factors. Following discussions the web-based calculator is now embedded in the web-page of a local charity and assessable for the general public and a slightly modified version integral part of the local e-referral system for colonic imaging. Further research into risk factor calculation based on evidence-based recommendations, empowering of the patient and expansion into other diseases is planned.
